# Soft multistable magnetic-responsive metamaterials

**DOI:** 10.1126/sciadv.adu3749

**Published:** 2025-07-16

**Authors:** Taylor E. Greenwood, Brian Elder, Md. Nahid Hasan, Jared Anklam, Saebom Lee, Jian Teng, Pai Wang, Yong Lin Kong

**Affiliations:** ^1^ Department of Mechanical Engineering, Rice University, Houston, TX 77005, USA.; ^2^ Department of Mechanical Engineering, University of Utah, Salt Lake City, UT 84112, USA.; ^3^ Rice Advanced Materials Institute, Rice University, Houston, TX 77005.

## Abstract

The wireless actuation of magnetic soft architectures can enable complex functionalities important in biomedicine and soft robotics. However, transforming and maintaining a device’s desired geometry without a sustained energy input remains challenging, especially where environmental stresses can be unpredictable. Here, we create a soft multistable magnetic-responsive metamaterial with programmable energy barriers enabled by a bistable geometry made entirely from soft material. The multistability and magnetic programming enable the soft metamaterials to reversibly transform between stable states, even under mechanical and thermal stresses that far exceed physiological conditions. In addition, the metamaterials can sustain compressive loads more than 10 times their mass, achieve shape reconfiguration in remote and confined spaces, and wirelessly deliver fluids against pressure, suggesting a broad range of future biomedical and soft robot applications.

## Teaser

An entirely soft, multistable metamaterial remotely transforms between stable states even when under environmental stress.

## INTRODUCTION

The wireless actuation of soft materials is of substantial interest in the fields of soft robotics and biomedicine. Magnetic fields can safely and efficiently travel through human tissue and have been used in clinical settings, such as in magnetic resonance systems with field strengths up to 8 T (*1*). Magnetic fields have been shown to be capable of remotely actuating and controlling magnetic soft biomedical devices within the complex and confined spaces of the human body (*2*). Recent advances have demonstrated magnetic-responsive soft architectures for implantable (*3*–*6*) and ingestible systems (*5*, *7*–*10*), where the rationally designed geometry and magnetic programming enable tailorable deformations, and the soft materials improve the safety and durability by reducing the device-tissue mechanical mismatch. In the field of soft robotics, magnetic soft constructs have been used to achieve locomotion (*4*–*7*, *10*, *11*) and as shape-morphing micromachines (*12*, *13*), arms and grippers for robots (*14*–*17*), miniature soft machines (*18*–*21*), reconfigurable antennas (*16*), and electromagnetic or acoustic filters (*22*, *23*). However, despite the impressive advances, maintaining multiple stable geometrical configurations without sustaining the input energy remains challenging, especially for biomedical devices where mechanical, chemical, and thermal stresses can be unpredictable and difficult to control.

In principle, an architecture can be designed to maintain transformed configurations without additional external energy input or control, and even against environmental stress, by incorporating a multistable geometry (*24*). However, creating an entirely soft multistable architecture that can be transformed by external magnetic fields remains challenging due to the competing objectives of stability and deformability. The majority of previous magnetic soft constructs are not multistable and require a sustained magnetic field (*3*, *5*, *10*–*13*, *18*, *20*, *21*, *25*–*28*) or temperature/light modulation (*4*, *14*, *16*, *22*, *23*) to maintain the transformed configurations, which inherently constrain the potential applications. While some previous demonstrations have included both magnetic field–induced transformation and multistability, they did not demonstrate the ability to maintain stable states under externally applied loads (*29*–*31*), require relatively high modulus materials to achieve the desired boundary conditions (*29*, *30*, *32*–*35*), or require stiff plates and sharp features (*7*, *15*, *17*), which are undesirable for biomedical applications. For example, for an ingestible or implantable device, stress concentrations are associated with gastric ulcers (*36*, *37*), gastric puncture (*38*), inflammation (*37*, *39*, *40*), and restenosis (*40*, *41*). An entirely soft multistable architecture capable of magnetic field–induced transformation between stable states is desired for a broad range of biomedical applications such as implantable devices, ingestible systems, and soft robots but has yet to be demonstrated.

**Fig. 1.**
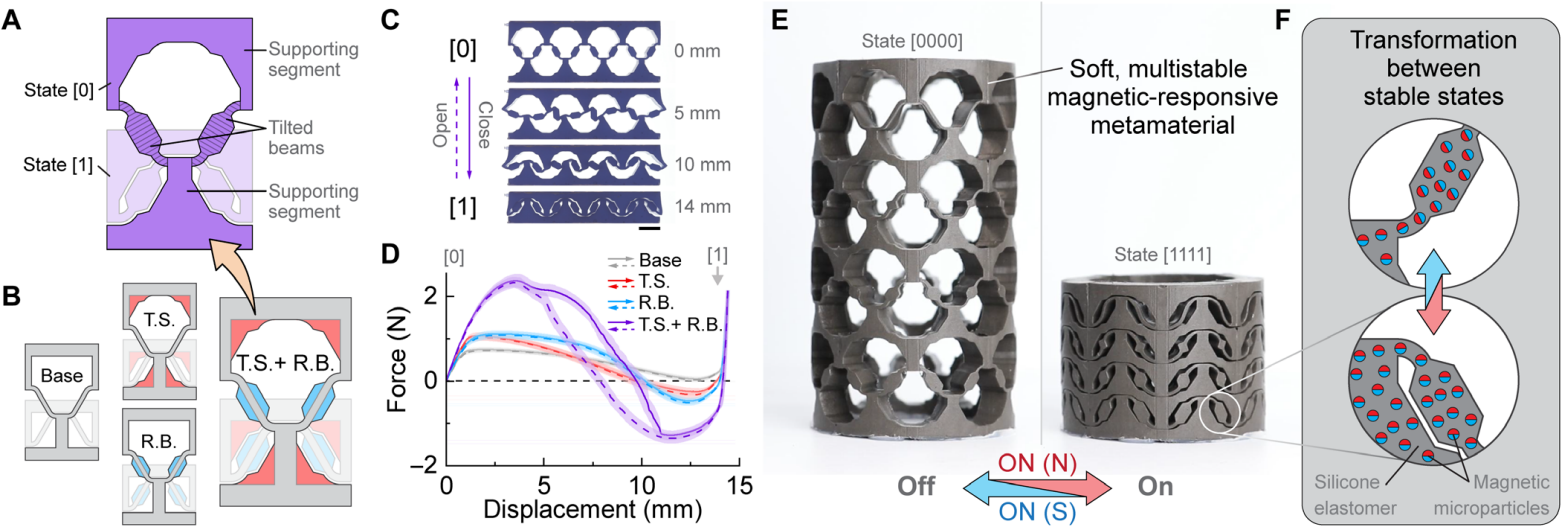
Soft, multi-stable metamaterials. (**A**) A soft multistable metamaterial unit cell is designed to be stable in the initial configuration (denoted as “[0]” state) and the transformed configuration (denoted as “[1]” state) and is composed of tilted beams (crosshatched) and supporting segments (solid). In (A) and (B), state [1] is lighter in color. (**B**) The multistable metamaterial can be created entirely from soft material by adding trapezoidal supporting segments (T.S., red) and reinforced beams (R.B., blue) to a base unit cell geometry (base, gray). (**C**) Four images during loading in compression of a metamaterial that has one row of four unit cells (1 × 4, row × column) with the T.S. + R.B. unit cell geometry (images for 1 × 4 metamaterials with the other unit cell geometries are shown in fig. S2). In the images, the metamaterial transitions between the open state [0] and the closed state [1] when a vertical load is applied. Scale bar, 10 mm. (**D**) Experimental force-displacement curves in compression (solid) and tension (dashed) for the four different 1 × 4 metamaterial designs, each composed entirely of one type of unit cell. The lines indicate the mean and shaded regions around the data show one SD for 16 experiments (two models with eight tests each) for each geometry. (**E** and **F**) Reversible transformation between stable states is enabled by creating the soft metamaterial from a ferromagnetic composite soft material. In (E), images show a four-row cylindrical metamaterial that geometrically transforms with a permanent magnet (on). The transformed configurations are maintained even when the magnet is removed (off) due to the multistable geometry. Dimensions are 30.2-mm outer diameter and 54.8-mm height in expanded state [0000]. The schematic in (F) shows the programmed magnetic microparticles within the soft material that enable remote and reversible transformation between stable states by a nonuniform field.

To create a soft, multistable magnetic-responsive metamaterial, here we design a bistable geometry made entirely from soft silicone and program the material’s magnetic domain to enable rapid and reversible transformation between stable states (Fig. 1). The metamaterial geometry’s energy barriers enable the metamaterial to maintain stable states even under environmental stress and dynamic perturbations and can be programmed on the basis of target applications. The magnetically programmed metamaterial enables wirelessly actuated transformations between stable geometric configurations that are critical for applications in confined or remote environments such as within the body. The entirely soft metamaterial demonstrates exceptional flexibility, chemical resistance, and thermal stability that can be leveraged to create soft devices such as reconfigurable actuators, volume-adjustable ingestible systems, and wirelessly actuated flow control assemblies capable of preserving their multistability and transformation abilities even when environmental conditions (e.g., temperature) and external stimuli (e.g., impacts) are challenging to predict or control.

## RESULTS

### Soft multistable magnetic-responsive metamaterials

To create a multistable mechanical metamaterial entirely from soft material, we build upon a bistable unit cell composed of two tilted beams connected by supporting segments. The unit cell is designed to transform between the initial stable configuration (denoted as state “[0]”) and the transformed stable configuration (denoted as state “[1]”) by loading in the vertical direction. As shown in fig. S1, a bistable unit cell will have a force-displacement curve that crosses below zero and an energy curve with a local minimum at state [1]. The force barriers to transition between states are calculated as the maximum and minimum of the force curve between the stable states, and the energy barriers are calculated as the difference between adjacent minima in the energy curve (see fig. S1). In principle, stability in state [0] is achieved by fabricating the unit cell in the initial configuration without residual stresses, while stability in state [1] is achieved by constraining the bending behaviors of the unit cell to prevent the tilted beams from returning to their undeformed orientations. However, achieving stability in state [1] is challenging with an entirely soft design due to deformations of the soft (i.e., low modulus) material from the nonzero stresses in the transformed state. One-dimensional (1D) bistable elements such as the tilted beam and von Mises truss are typically represented with rigid boundary conditions (*42*, *43*) that constrain the bending behaviors to enhance stability but are incompatible with an entirely soft design. Similarly, previous multistable silicone architectures with tilted beams require materials with a relatively high modulus (Young’s modulus, *E* ≈ 2 GPa) in the supporting segments to constrain the unit cell motion (*29*, *30*, *32*–*35*, *42*, *44*).

Here, we resolve the previous need for relatively high-modulus materials in the supporting segments by designing an entirely soft (*E* ≈ 800 kPa) multistable metamaterial with trapezoidal supporting segments (“T.S.”) and reinforced beams (“R.B.”) (Fig. 1, A to D). To enable relatively higher force and energy barriers in compression than tension, we choose to fabricate the metamaterial in the fully expanded configuration, as residual stresses from deformation have been shown to decrease the force and energy barriers required to transition a bistable metamaterial from the deformed configuration to the fabricated configuration (*42*). As shown in Fig. 1D and fig. S2, the strategic geometry modifications increase the energy barriers by constraining the unit cell’s bending behaviors without changing the unit cell size or soft material. The hysteresis in the force curves and increased energy barriers for the T.S. + R.B. unit cell geometry can be attributed to the highly constrained unit cell motion. For example, during the transformation, we observe that the unit cell’s beams become temporarily locked in orientations that cause compression of the reinforced sections of the beams (e.g., indicated by red circles in fig. S2, D and E). As loading continues, a portion of the elastic energy stored in the compressed beams is dissipated as the vertical component of the compression changes direction due to the rotation of the beam. The increased stability is synergistic with the energy barrier in tension (*E*_tens_), which is more challenging to achieve due to the nonzero stresses in the state [1]. For example, a metamaterial with a combination of T.S. and R.B. has *E*_tens_ = 6.2 ± 0.7 mJ (mean ± one SD), which is substantially higher than the *E*_tens_ for metamaterials with only T.S. or R.B. alone (0.9 ± 0.4 and 1.3 ± 0.3, respectively; fig. S2 and table S1). The high-energy barriers in both loading directions are highly desirable for maintaining multiple stable configurations in dynamic environments and under external pressure without external energy input or modulation of environmental factors (e.g., temperature).

To enable remotely-actuated transformation between stable states, we create the soft metamaterial from a ferromagnetic composite material consisting of silicone elastomer and hard-magnetic NdFeB (neodymium-iron-boron) microparticles and program the magnetic remanence parallel to the transformation direction when in the collapsed state (Fig. 1, E and F). Specifically, the transformation from state [0] to [1] is achieved by applying an external nonuniform magnetic field below the metamaterial to cause forces and torques that close the unit cells into state [1], and the transformation from state [1] to [0] is achieved by applying the magnetic field with reversed polarity to cause forces and torques that open the unit cells into state [0]. The soft, magnetic-responsive metamaterial not only transforms when actuated by a nonuniform magnetic field (“on”) but also maintains the transformed configurations even after the field is removed (“off”) due to the geometric multistability. In contrast, prior magnetic soft constructs were not multistable and required a sustained magnetic field (*3*, *5*, *10*–*13*, *18*, *20*, *21*, *25*–*28*) or temperature/light modulation (*4*, *14*, *16*, *22*, *23*) to maintain transformed states.

### Programming stability with the geometrical design of the unit cell

To enable selective actuation and tailorable energy barriers based on target applications, we program the energy barriers by adjusting the geometrical design of the unit cell. Specifically, we tune the stability of bistable unit cells by modulating the beam’s thickness and angle (*42*) and by reinforcing the beam’s midsection (*45*). In contrast to the previous works that required materials with a relatively high modulus in the supporting segments, our approach enables programmable multistability while preserving the metamaterial’s entirely soft design.

**Fig. 2.**
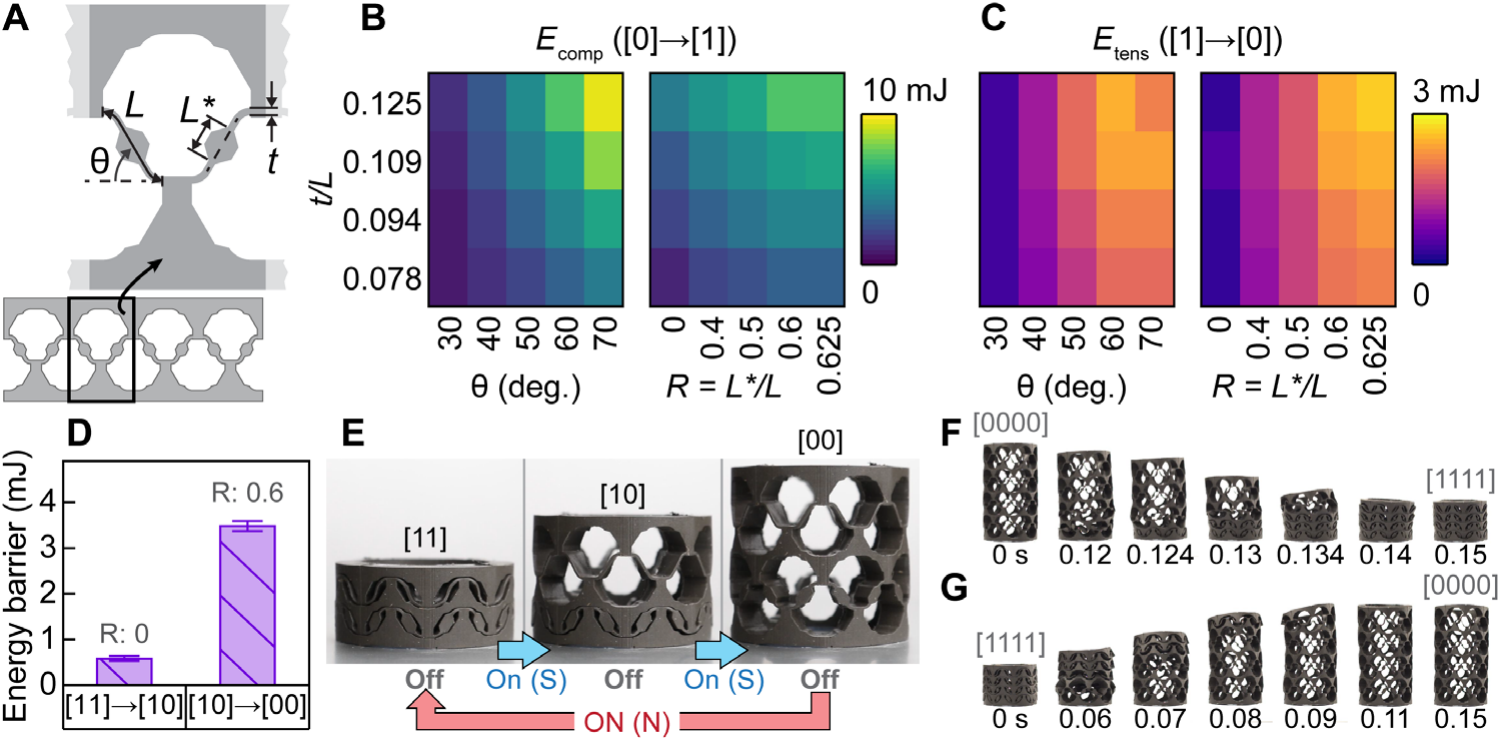
Programming stability with beam geometry of the unit cell. (**A**) Schematic shows a single unit cell of the 2D 1 × 4 model used in the FE simulations. (**B** and **C**) Energy barriers in compression (B) and tension (C) due to changes in *t*/*L*, θ, and *R*. In plots with varying *R*, θ = 60°. Likewise, in plots with varying θ, *R* = 0.6. (**D**) Experimental energy barriers in tension for a 2 × 8 cylindrical metamaterial, where the stability is programmed by changing *R* in each row. Bars indicate the mean values, and error bars show one SD across five tests. (**E**) Ability to program the energy barriers enables magnetic field–induced transformation of sequential rows of the 2 × 8 cylinder. Dimensions: 30.2-mm outer diameter and 29.1-mm height in fully expanded state [00]. (**F** and **G**) High-speed images show the rapid contraction (F) and expansion (G) of a 4 × 8 cylindrical metamaterial with selectively programmed energy barriers (where *R* = 0.6, 0.4, 0.25, and 0 from bottom to top row) to enable complete expansion. Dimensions: 30.2-mm outer diameter and 54.8-mm height in fully expanded state [0000].

We use finite-element (FE) simulation to explore the design space while maintaining identical material properties and boundary conditions (Fig. 2, A to C). The FE simulations are performed in Abaqus on 2D 1 × 4 (row × column) metamaterials without contact interactions (details in Materials and Methods). 2D simulations are chosen to reduce the high computational cost from the geometric nonlinearities (which increase with the changes to the unit cell that enhance the bistability, as shown in Fig. 1, A to D), and the instabilities inherent in the motion between stable states. Because of the highly constrained motion that enables the high energy barriers, the 3D experimental models exhibit some out-of-plane bending that reduces the constraints on the motion during transformation. However, in simulations, including contact interactions locks the metamaterial’s motion when different segments of the metamaterial contact each other, making it challenging to evaluate the transformation behavior. Thus, to reduce the constraints on the motion while preserving the model’s dominant behavior, 2D simulations exclude contact interactions. A comparison between experiment and FE simulation results in fig. S3 indicates that the simulations capture the dominant behaviors of the metamaterials and the differences in stability between the designs (fig. S3 and tables S1 and S2). Because of the assumptions in the FE simulations, the results in Fig. 2 (B and C) are intended as qualitative predictions of the effect of changing the unit cell geometry on the energy barriers of the soft metamaterial. The unit cell geometry is adjusted by varying the beam slenderness ratio *t*/*L*, beam angle θ, and beam reinforcement ratio *R*, where *t* is the thickness of the unreinforced beam, *L* is the beam length (6.4 mm), L* is the length of the reinforced section, and *R* = L*/*L*. Dimensions of the unit cell are included in Fig. 2A and fig. S4. For the beam angle, we focus on higher values of θ that enable larger expansion ratios (assuming a constant thickness of the horizontal connecting portions of the supporting segments, see fig. S4), where the upper limit of θ = 70° is designed to prevent backward rotation of the beams during loading. For the beam reinforcement ratio, we evaluated *R* at values of 0, 0.4, 0.5, 0.6, and 0.625. We focused on designs with higher values of *R* that we hypothesized would have higher energy barriers (for this particular design, *R* = 0.625 is the upper limit if the reinforced section only occurs within the linear section of the beam) while also including the design without beam reinforcement (i.e., *R* = 0) to understand the range of energy barriers that could be achieved with a given *t*/*L* and θ.

The simulation results in Fig. 2 (B and C) qualitatively demonstrate that adjusting the geometric design of the unit cell enables large and predictable variations in the magnitude of the energy barriers in both loading directions for 1 × 4 metamaterials. Specifically, Fig. 2B shows that the energy barriers in compression tend to increase with increasing *t*/*L*, θ, and *R*. The consistency of the qualitative trends in Fig. 2B with prior literature (*42*, *45*) suggests that the reduced-order simulations were capable of capturing the dominant behaviors that influence the bistability. Results in Fig. 2C show that the energy barriers in tension generally increase with increasing *t*/*L*, θ, and *R*. As anticipated, the energy barriers in tension (Fig. 2C) are lower than the energy barriers in compression (Fig. 2B) due to the residual stresses from the deformation. The results in Fig. 2 (B and C) can be leveraged to program metamaterial unit cells based on target applications, where the wide range of energy barriers can enable a broad range of potential applications.

Modulating *t* and *R* is advantageous for metamaterial design, as the unit cell width and height are unaffected by the geometry changes. In fig. S5, we also demonstrate that the entirely soft metamaterial can transition between stable states even with *R* that is 8 to 11% larger than the kinematic feasibility limit for a bistable unit cell with reinforced beams (*45*), where the ability to surpass the kinematic feasibility limit is enabled by the deformability of the entirely soft design. Because of the strong effect of increasing *R* on increasing the energy barriers in both loading directions, the ability to design the metamaterials with higher values of *R* can be leveraged in future work to further increase the energy barriers of soft devices for target applications.

The results in Fig. 2 (B and C) suggest that by strategically programming the energy barrier of the individual unit cell, we can create a soft structure that selectively responds to a given magnetic field input. We experimentally demonstrate this result in Fig. 2 (D and E) with a 2 × 8 cylindrical metamaterial with *R* = 0 in the top row and *R* = 0.6 in the bottom row. As shown in Fig. 2E, the programmed stability enables the metamaterial’s top row to be selectively triggered to transform from states [1] to [0] before the bottom row using a relatively lower field strength (e.g., from nonuniform field actuation) (table S3). Similarly, as shown in Fig. 2 (F and G), we can also program the rows of a 4 × 8 cylindrical metamaterial with a range of energy barriers (where *R* = 0.6, 0.4, 0.25, and 0 from bottom to top row) to enable full expansion and contraction of the metamaterial. In contrast, the structure cannot be fully expanded with the same magnetic field input if it is designed with *R* = 0.6 in each row due to its higher force barriers (fig. S6, table S4, and movie S1). The high-speed video images in Fig. 2 (F and G) demonstrate the metamaterial’s rapid contraction and expansion within 0.15 s when actuated by the nonuniform field (movie S2) at a comparable speed with previous demonstrations (*13*). Also, as described earlier, in contrast to the previous demonstrations (*3*, *5*, *10*–*13*, *18*, *20*, *21*, *25*–*28*) that require an applied magnetic field to maintain the transformed state, our metamaterial remains stable even after turning off the magnetic field.

### Metamaterial performance and potential applications

**Fig. 3.**
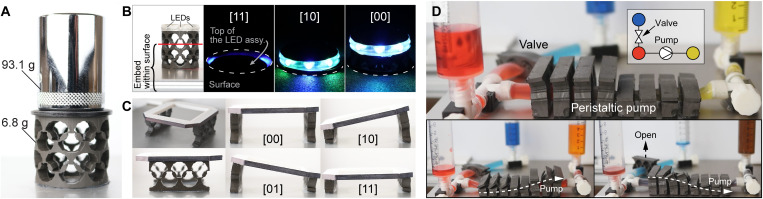
Metamaterial performance and potential applications. (**A**) Programmed energy barriers enable a 2 × 8 cylindrical metamaterial to maintain the open configuration without an external magnetic field even under load from 13.7 times its mass. (**B**) The programmable energy barriers and remote actuation are leveraged to deploy a metamaterial with integrated LEDs between multiple stable configurations in a confined space. In images 2, 3, and 4 in the series, the metamaterial and LED assembly are confined within a surface with only the top exposed to demonstrate shape reconfiguration in confined spaces. (**C**) Magnetic programming and nonuniform field actuation enable selective transformation of metamaterials, as demonstrated by a tilting stage. (**D**) Multistability and selective triggering enable the remote actuation of 1 × 3 metamaterials that form a valve and peristaltic pump that remain stable when the magnetic field is removed even against the fluid pressure from pumping. In the bottom left inset, the red fluid is pumped to the yellow reservoir. In the bottom right inset, the valve is opened, and the blue fluid is then pumped to the yellow reservoir.

The metamaterial’s geometric multistability, programmable energy barriers, nonuniform magnetic field actuation, and soft design can enable a broad range of potential applications (Fig. 3). For example, in Fig. 3A, we show that the programmed energy barriers enable a 2 × 8 cylindrical metamaterial to maintain its open configuration without an externally applied magnetic field even under a compressive load from 13.7 times its mass, suggesting future applications where stability against compression is desirable. In Fig. 3B, we show a metamaterial integrated with a light-emitting diode (LED) assembly to highlight two potential functionalities enabled by the metamaterial (movie S3). First, the metamaterial’s magnetic field–induced transformation enables reconfiguration in confined spaces, as shown in the darker images where only the top surface is exposed. In the images, the light from the LEDs enables visualization of the metamaterial even when fully embedded in the surface when in state [11]. The ability to transform within a confined space can be leveraged in future biomedical applications, such as reconfigurable ingestible or implantable devices that can be transformed by remote magnetic fields and maintain multiple stable volumes and shapes even in the absence of an actuating field. Second, the selectively programmed energy barriers can enable rapid and remove deployment of tools (e.g., lights), measurement instruments, and other subassemblies between multiple programmed configurations. Building on this demonstration, we envision the ability to create future deployable instrumented architectures by integrating the metamaterial with conductive traces and off-the-shelf electronics (e.g., microprocessors). For example, a magnetic soft multistable metamaterial can enable a reconfigurable antenna that can rapidly deploy between multiple programmed configurations from a confined space.

In addition, the metamaterial can be built to achieve complex motions and functionalities that leverage the selective actuation capabilities of the nonuniform magnetic fields. In Fig. 3C, we show the ability to produce bending motions by combining the 1D linear transformation of different metamaterials (movie S4). As shown in the images, the metamaterial segments maintain their configurations when the assembly is in states [10] and [01] even while bending out of plane, which is enabled by the metamaterial’s high multistability and entirely soft design. This demonstration suggests that more complex motions (e.g., bending and twisting) can be achieved in future metamaterials by aligning the unit cell’s transformation direction to be parallel to the intended direction of motion. For example, in fig. S7, we show how 3D transformation of a sphere-like structure can be achieved by aligning metamaterials along the edges of a regular octahedron. Similarly, a wide range of metamaterial geometries (e.g., 3D constructs) can be created by strategically arranging the unit cells as shown in previous reports (*42*). As shown in Fig. 3D, we demonstrate the ability to robustly achieve the rapid and coordinated motions of a wirelessly controlled valve and peristaltic pump (movie S5). The peristaltic pump is created from eight separate 1 × 3 metamaterials (“pump segments”) with a compliant tube placed through the central hole of each pump segment. The pump is designed such that fluid can flow when a segment is in state [0] but cannot flow when the segment is in state [1]. Peristaltic pumping is performed by transforming the segments in a periodic motion to move the fluid between reservoirs. Experiments on the peristaltic pump show that, due to the multistability of the pump segments, the peristaltic pump is able to maintain zero flow rate up to a pressure of 6.4 kPa (equivalent to 65 cm H_2_O). Further, the pump can resist a pressure of 14.1 kPa (144-cm H_2_O) before any segment expands into state [0] and 18.5 kPa (189-cm H_2_O) before all segments expand into state [0]. In this demonstration, we achieve the selective transformation of individual segments by leveraging the nonuniform magnetic field and maintain transformed configurations even against the fluid pressure during pumping. This demonstration highlights the ability of our design to achieve complex motions and robustly maintain stability, in stark contrast to earlier multistable metamaterials that are unable to achieve the remote and selective actuation (*42*, *44*–*47*) and earlier magnetic soft constructs that are incapable of maintaining the transformed configurations without a constant magnetic field (*3*, *5*, *10*–*13*, *18*, *20*, *21*, *25*–*28*) or temperature change (*4*, *14*, *16*, *22*, *23*) for applications such as fluid pumping. In summary, the ability of the soft multistable metamaterial to selectively transform between multiple programmed stable configurations with a nonuniform magnetic field, as demonstrated with the representative examples in Fig. 3, can lead to a myriad of applications in soft robotics and biomedical devices.

To investigate the effect of the metamaterial size on multistability, we performed experiments on 1 × 3 metamaterials with the geometry scaled uniformly in all directions (fig. S8 and table S5). As shown in fig. S8, the experiment results indicate that the force barriers scale with size by the power of ~2 and the energy barriers scale with size by the power of ~3. These results align closely with analytical predictions of force scaling with size by the power of 2 and energy scaling with size by the power of 3 for small-length compliant flexures (details in the Supplementary Materials). The scaling relations also show that the flexure stiffness scales linearly with the elastic modulus, suggesting that the force and energy barriers will scale linearly with the elastic modulus. By following these scaling relationships, the metamaterial design should theoretically be scalable across a broad range of sizes, including at sizes smaller than presently demonstrated. Compliant mechanisms, which rely on the bending of flexible segments, are easily scaled and have even been experimentally demonstrated at the microscale (*48*). As the technical limitations to scaling the present metamaterial are primarily due to the casting fabrication, advances in fabrication (e.g., additive manufacturing) could facilitate scaling the metamaterial for both larger and smaller target applications.

### Multistability and transformation in dynamic environments

**Fig. 4.**
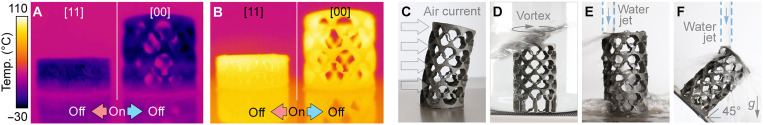
Metamaterial stability across temperature range and mechanical pressure. (**A** and **B**) Infrared imaging demonstrates the ability to transform and maintain stable states even in (A) cold (−20°C) and (B) hot (100°C) conditions. (**C** to **F**) 4 × 8 cylindrical metamaterial with differing energy barriers in each row can resist the pressure exerted by an air current at 6.6 m/s (C), a water vortex at 150 rpm (D), a vertical water jet at 97 ml/s (E), and a 45° water jet at 84 ml/s (F).

To evaluate the robustness of the metamaterial’s multistability in dynamic environments, cylindrical metamaterials are subjected to temperature changes and mechanical pressure (Fig. 4). In Fig. 4 (A and B) and movie S6, we show the metamaterial’s magnetic field–induced transformation and stability between approximately −20° to 100°C, which would not be feasible for previous magnetic soft architectures that require shape memory polymers and temperature change to maintain the transformed configurations (*4*, *14*, *16*, *22*, *23*). The metamaterial’s ability to transform and maintain stable states across temperatures that far exceed physiological conditions (−20° to 100°C) can enable applications where the metamaterial remains in the programmed configuration even during temperature fluctuations in environments where temperature may be challenging to predict or control.

To maintain stable states in dynamic environments, we can strategically program the energy barriers to be higher than the anticipated level imposed by stressors but remain lower than the energy input applied by the magnetic field for transformation. As shown in Fig. 4 (C to F) and movie S7, we demonstrate the ability of the soft structure to withstand a wide range of environmental stressors such as air current (6.6 m/s), a water vortex (150 rpm), and water jets (vertical and at 45° with flow rates of 97 and 84 ml/s, respectively). For example, the bottom two rows of the 4 × 8 metamaterial can be expanded with a magnetic field even when under constant impingement from the vertical water jet. In principle, the metamaterials should be able to maintain their stable state indefinitely without external magnetic stimuli (or disruption from other stimuli) that exceed the energy and force barriers of the metamaterial, which is consistent with our experimental observation.

### Multistability and transformation after adverse stressors

**Fig. 5.**
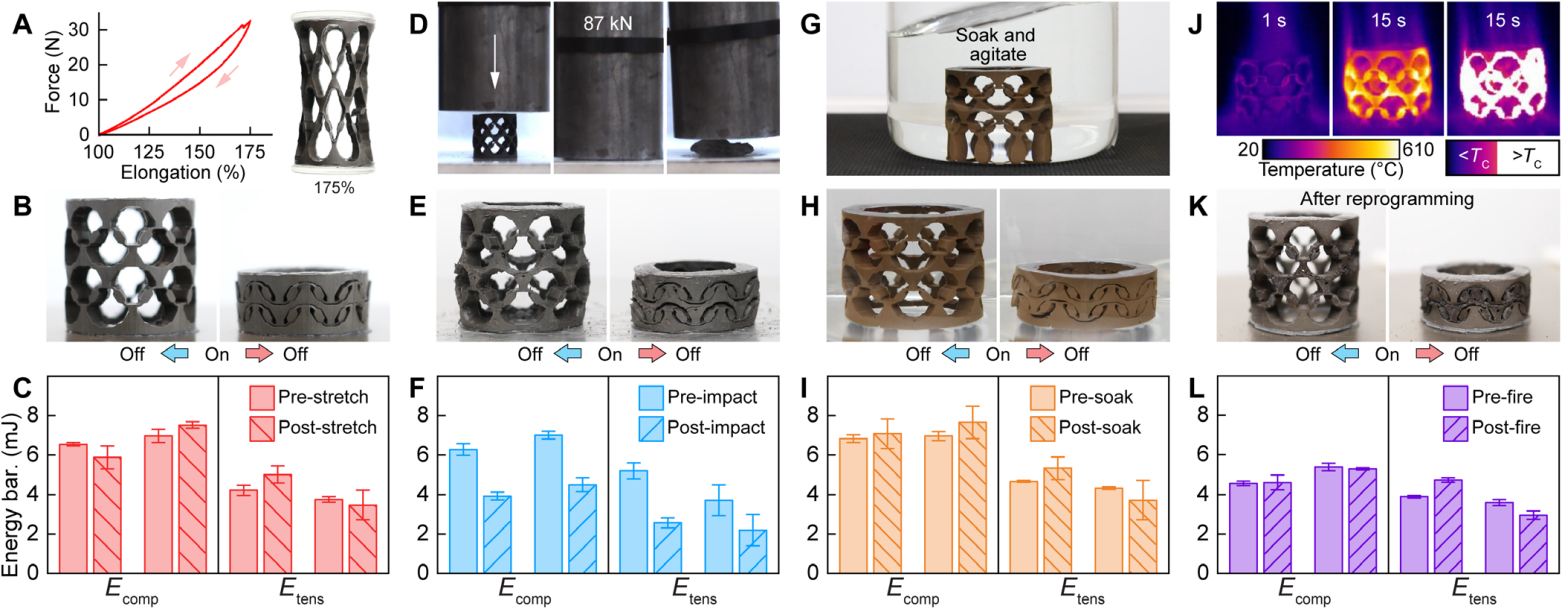
Metamaterial recovery after mechanical, chemical, and thermal stressors. The 2 × 8 cylindrical metamaterials are able to recover their multistability and transformation abilities even after extensive stretching to 175% (**A** to **C**), a rapid blunt impact with a force of 87 kN (**D** to **F**), a week-long exposure to acid (simulated gastric fluid) (**G** to **I**), and fire exposure for 15 s (**J** to **L**). In (B), (E), and (H), the metamaterial is able to transform between stable states with an external magnetic field after each stress, respectively. In (K), the transformation capability after the thermal stressor was fully restored after reprogramming the magnetic microparticles. In (C), (F), (I), and (L), experimental data show the metamaterial energy barriers of each case, respectively, where the bars represent the mean and error bars show one SD across five tests.

We further demonstrate the metamaterial’s ability to recover functionality after adverse mechanical, thermal, and chemical stressors (Fig. 5). The entirely soft metamaterial can effectively achieve geometrical transformation and maintain stable states without requiring materials with a relatively high modulus in the supporting segments, which reduces susceptibility to fracture, plastic deformation at relatively low strains, material degradation, and failure at material interfaces.

For example, the entirely soft and elastomeric metamaterial can robustly withstand high mechanical strain, in contrast to the previous bistable and multistable magnetic architectures. As shown in Fig. 5A and movie S8, the 2 × 8 cylindrical metamaterial can endure high localized strain (ε ≈ 1.40) within the thin sections of the beams while being uniaxially stretched to 175% of its initial height (ε = 0.75), where only one beam is fractured during the loading-unloading cycle. After stretching, the metamaterial remains capable of transforming between stable states with an external magnetic field (Fig. 5B and movie S8), where the energy barriers change by −10 to +19% (Fig. 5C and table S6; percentages indicate the difference between average values across five experiments). The ability of the metamaterial to preserve its multistability after high strain is due to the entirely soft design, the inherent material properties of silicone elastomers that can endure high strain before failure, and the redundancy of the multiple unit cells in each row of the metamaterial.

In addition, we evaluate the effects of extreme bending and compression on metamaterial’s performance. For instance, we apply a rapid blunt impact with a resultant force of 87 kN to a 2 × 8 cylindrical metamaterial with an 8 kg of cylinder dropped from 1.5 m. As shown in Fig. 5D and movie S9, the entirely soft design enables the metamaterial to flatten to ~10% of its original height under extreme and rapid impact. Despite the physical damage from an impact force that was four orders of magnitude greater than the metamaterial’s programmed force barriers (*F*_comp_ for [00] to [10] = −1.43 ± 0.04 N), our structure preserves both its multistability (where the energy barriers only decrease by 36 to 51%; Fig. 5F and table S6) and transformation abilities (Fig. 5E) without beam breakage. The ability of the metamaterials to recover their functionality even after extreme forces and physical damage (which is due to the entirely soft design, the inherent material properties of the silicone, and the redundancy of the multiple unit cells in each row) suggests potential applications in dynamic and adverse environments.

We also evaluate the impact of prolonged chemical exposure, which can expand the metamaterial’s potential applications to implantable or ingestible systems [e.g., exposure to gastric fluids for gastric resident devices (*49*) and electronics (*50*)]. Here, we show that a 2 × 8 cylindrical metamaterial remains functional after immersion in simulated gastric fluid for 7 days (Fig. 5, G and H, and movie S10, at room temperature and 120 rpm in a shaker), with a small impact (<15%) to its programmed energy barrier (Fig. 5I and table S6). The ability to recover after exposure to acid is due to the inherent material properties of silicone elastomers, which have been previously used to encapsulate gastric devices (*51*). This demonstration highlights one of the key advantages of our work, as we have created the multistable metamaterial entirely from the soft silicone. To the best of our knowledge, no other magnetic-responsive metamaterial has been demonstrated to maintain transformation abilities after extended exposure to simulated gastric fluid. Our approach can complement metamaterial designs that rely on chemical exposure to trigger transformation between states (*42*, *46*) in a broad range of applications that subject the device to chemical exposure, such as with ingestible devices, suggesting applications of using the metamaterials as transpyloric stents to treat gastroparesis (*52*) or triggerable gastric distention to induce satiety in the treatment of obesity (*49*).

Last, we examine the structure’s robustness in extreme thermal stress. Specifically, we engulf a 2 × 8 cylindrical metamaterial in flames for 15 s (Fig. 5J and movie S11) and show that the structure can maintain its multistability (with the energy barriers changing by −18 to +21%; Fig. 5L and table S6) and recover its ability to transform by external magnetic fields (Fig. 5K and movie S11). We note that, as anticipated, reprogramming of the magnetic domain is needed to fully restore the transformation abilities, as large portions of the metamaterial exceeded the Curie temperature (*T*_C_ = 306°C), which causes demagnetization of the magnetic microparticles. The entirely soft design, the inherent material properties of the silicone, and the redundancy of the multiple unit cells in each row enable the metamaterial to preserve its robust multistability even after the fire exposure caused physical damage due to the temperatures that exceeded the silicone’s service temperature range. In contrast, previous metamaterials that were not entirely created from silicone may undergo irreversible material degradation (e.g., melting) from exposure to fire or high temperatures.

## DISCUSSION

In this study, we developed an entirely soft, multistable, magnetic-responsive metamaterial that can be transformed between programmable stable states while maintaining high-energy barriers. The metamaterial can be remotely actuated with external magnetic fields to transform between stable configurations across a range of temperatures that far exceed physiological conditions and can retain the transformed configuration under load, even after the magnetic field is removed. We also showed that the metamaterial can be programmed to selectively respond to a given magnetic field by adjusting the energy barrier of each unit cell and demonstrated complex transformations with potential applications in ingestible biomedical devices. These properties are highly desirable for a wide range of biomedical applications, such as reconfigurable implantable and ingestible devices.

To systematically address how the present metamaterial overcomes the limitations of previous studies, we first showed that unit cell design enables the magnetic metamaterial to maintain stable states even after the actuating magnetic field was removed, in contrast to previous magnetic soft constructs that could not maintain transformed states without a sustained magnetic field (*3*, *5*, *10*–*13*, *18*, *20*, *21*, *25*–*28*). Next, we showed the metamaterial could be transformed and maintain stable states between −20° and 100°C, in contrast to previous magnetic soft constructs that required temperature change to maintain the transformed states (*4*, *14*, *16*, *22*, *23*). Last, in contrast to previous multistable constructs that included relatively high modulus materials or multiple materials (*29*, *30*, *32*–*35*, *42*, *44*), we showed that the entirely soft design and the inherent material properties of the silicone enabled the metamaterial to preserve its multistability and magnetic field–induced transformation after high mechanical strain, extreme bending, and compression. We observed a limitation in its ability to transform after fire exposure due to the magnetic particles’ demagnetization from the high temperatures, which was remedied by reprogramming the magnetic remanence. Notably, the metamaterial maintained its multistability and transformation ability after prolonged exposure to simulated gastric fluid, highlighting its applicability for ingestible medical devices.

## MATERIALS AND METHODS

### Model fabrication

The multistable mechanical metamaterials were fabricated from silicone elastomers in a casting process using 3D printed molds (Form 3/3+ printer and Clear Resin, Formlabs). The material was prepared silicone rubber (Dragon Skin 30, Smooth-On). For the magnetic metamaterial, the silicone part A was first mixed with NdFeB magnetic microparticles (Magnequench) and a cure accelerator (Plat-Cat, Smooth-On) for 45 s in a planetary centrifugal mixer (AR-100, Thinky) before mixing with the silicone part B for 30 s at room temperature. After mixing, the silicone was cast in the molds, cured, and demolded. Magnetic programming was performed by deforming the specimen into state [1] and then programming with an impulse magnetizer (IM-10-30, ASC Scientific) at 2 T. The geometry and dimensions of the metamaterials used in this study are provided in table S7.

### Tensile testing

Metamaterial specimens were analyzed on a benchtop uniaxial tensile tester (Tinius Olsen) with a 250-N load cell. Tensile tests were performed with controlled displacement in a complete loading cycle at a strain rate of 10 mm/min. Tensile test results were compared with simulations using material properties from dogbones (ASTM D412-C geometry) created from the same batch of silicone as the experiment specimens. Dogbones were tensile tested by cycling the specimens between 0 and 80% strain at a strain rate of 150 mm/min. Because of variations in the actual strain rate during testing of the metamaterials, the raw data were resampled with a constant displacement step of 0.02 mm to enable calculations of the mean and SD at each displacement. The energy-displacement data were calculated by numerical integration along the force-displacement curve using the trapezoidal method. Force barriers were calculated by finding the critical points (i.e., maxima and minima) in the force-displacement data. Energy barriers were calculated by the difference between adjacent minima in the energy curves.

### Peristaltic pump pressure

The pressures that a peristaltic pump segment could resist with zero flow rate and before expanding into state [0] were determined using a column of water connected to the inlet of the peristaltic pump. Air was purged from the inlet, but the pump and outlet were left dry. The outlet was left exposed to atmospheric pressure. The water was fed via drip-feed and was colored to aid visualization. The water column reached a height of ~65 cm (equivalent to a pressure of ~6.4 kPa), before flow was observed at the pump outlet. Next, the pump outlet was closed, and water continued to be added. A water height of 144 cm (14.1 kPa) was reached before any pump segment expanded into state [0]. A water height of 189 cm (18.5 kPa) was reached before all pump segments expanded to state [0]. The pump’s maximum pressures could be increased by increasing the force barriers of the pump segments in state [1], which could be achieved by increasing *t*/*L*, θ, *R*, the unit cell size, and the material modulus.

### Magnetic actuation

Magnetic actuation was performed by positioning and moving a permanent magnet (K&J Magnetics) below the samples, similar to previous demonstrations (*13*), which can be automated using a robotic arm similar to earlier demonstrations (*6*). To actuate from state [0] to [1], the magnet was positioned below the metamaterial with the north pole toward the metamaterial. To actuate from state [1] to [0], the magnet was positioned below the metamaterial with the south pole toward the metamaterial.

### Imaging

Videos and images were captured with various cameras as follows: high-speed actuation (NX4-S3, IDT Vision), thermal tests (AX5, FLIR), tensile testing (EOS 80D, Canon), and all other videos and images (EOS 80D, Canon). Actuation speed measurements were calculated using the frame rate and number of frames between stable configurations.

### Adverse stressor experiments

The stretching experiment was performed on the uniaxial tensile tester using a 2 × 8 cylindrical metamaterial adhered to custom mounts. The specimen was pulled to 175% of its original height in a loading-unloading cycle, after which the magnetic field–induced transformation and stability were evaluated.

The blunt impact experiment was performed by dropping a metal cylinder (“impact body”) of 8 kg from a height of 1.5 m onto the 2 × 8 cylindrical metamaterial and aluminum plate set on flat concrete. To limit the impact body’s rotation, it was connected to a vertical aluminum rail with sliding mounts. The impact force was calculated using the impact body’s mass, velocity before and after impact, and impact time from the high-speed video. After the impact, the stability and magnetic field–induced transformation were evaluated.

The chemical exposure experiment was performed with a 2 × 8 cylindrical metamaterial inside a glass beaker. The beaker was filled with simulated gastric fluid (Ricca Chemical) and then placed on a digital orbital shaker at 120 rpm and room temperature for 7 days. After 7 days, the magnetic field–induced transformation was demonstrated, while the metamaterial remained within the fluid. The metamaterial was then removed, rinsed, and dried, and stability was measured.

The fire experiment was performed by dispensing isopropyl alcohol onto the 2 × 8 cylindrical metamaterial and then magnetically actuating the metamaterial into the [00] configuration. Next, the isopropyl alcohol was ignited to engulf the metamaterial in flames for ~15 s before the fire was extinguished. Transformation by external magnetic fields was attempted as the metamaterial cooled to room temperature. After cooling, the stability was measured, and the magnetic field–induced transformation was attempted. The magnetic field–induced transformation was demonstrated after reprogramming the magnetic particles with the impulse magnetizer.

### FE simulations

The FE simulations were performed in Abaqus (Dassault Systèmes) with geometry files that were previously created in computer-aided design software (SolidWorks, Dassault Systèmes) and saved as STEP files. The geometric nonlinearities of the design and instabilities inherent in the motion between stable states can result in FE simulations with high computational cost, particularly for simulations in three dimensions or with contact interactions. Consequently, we performed reduced-order simulations with 2D planar homogeneous solid models to reduce the computational complexity while preserving the model’s dominant behavior. To account for the out-of-plane bending that is challenging to capture in 2D simulations, no contact interactions were included. To account for the out-of-plane thickness of the experiment models, the simulation forces were multiplied by the thickness (5 mm) in postprocessing. The hyperelastic material model was created using a Marlow model created from uniaxial tensile test data from dogbones of the silicone material (fig. S9), with a density of 1.1 g/cm^3^ (value from the material datasheet) and a Poisson’s ratio of 0.5. The simulations used nonlinear geometry due to the high deformations in the unit cell motion and dynamic, implicit quasi-static analysis to improve convergence due to the instabilities inherent in the transformations.

The FE solver used a full Newton solution technique with a maximum of 10,000 increments and a minimum increment size of 1 × 10^−15^. For the simulations, the FE mesh was created with four-node bilinear plane strain quadrilateral hybrid elements (CPE4H) with a size of 0.1 mm (deviation factor = 0.04, minimum size = 0.04). The boundary conditions on the bottom edge of the geometry were fixed in the horizontal and vertical directions, while the boundary conditions on the top edge had horizontal displacement fixed. In the first simulation step, which simulates the transformation from state [0] to state [1], the top edge boundary condition had a vertical displacement greater than the height difference between the stable states. In the second simulation step, which simulates the transformation from state [1] to state [0], the top edge vertical displacement was set to zero. The force-displacement response was measured as the sum of the vertical reaction forces at every node on the top edge. To maintain consistency in the parameter study simulations, a custom Python code was used to control the FE setup, processing, and data export. For the simulation data in fig. S3, the simulation geometry profile boundaries were offset by −0.05 mm to compensate for horizontal expansion during the printing of the experiment specimen molds.
