# Supplementary material for: Soft multistable magnetic-responsive metamaterials
Source: Sci Adv. 2025 Jul 16;11(29):eadu3749. doi: 10.1126/sciadv.adu3749 (PMC12266116; doi:10.1126/sciadv.adu3749)
Supplement: Supplementary file 1 — Supplementary Text Figs. S1 to S9 Tables S1 to S7 Legends for movies S1 to S11 [file sciadv.adu3749_sm.pdf]

Science Advances
AAAS

# Supplementary Materials for

## Soft multistable magnetic-responsive metamaterials

Taylor E. Greenwood *et al.*

Corresponding author: Yong Lin Kong, kong@rice.edu

*Sci. Adv.* **11**, eadu3749 (2025)
DOI: 10.1126/sciadv.adu3749

**The PDF file includes:**

Supplementary Text
Figs. S1 to S9
Tables S1 to S7
Legends for movies S1 to S11

**Other Supplementary Material for this manuscript includes the following:**

Movies S1 to S11

**Supplementary Text**

Effect of size and modulus on metamaterial multi-stability.

To evaluate the effect of metamaterial size on multi-stability, we performed experiments with 1×3 metamaterials with the geometry scaled uniformly in all directions (fig. S8, table S5). The results indicate that the force barriers scale with size by the power of approximately 2 and the energy barriers scale with size by the power of approximately 3, which aligns closely with analytical predictions of force scaling with size by the power of 2 and energy scaling with size by the power of 3 for small-length compliant flexures.

For the analytical prediction, we choose to represent the segments that experience the most bending (i.e., the area between the reinforced sections of the beam and the supporting segments) as small-length compliant flexures due to their small length-to-thickness ratio. The Pseudo-Rigid-Body Model (48) predicts the torsional stiffness of a small-length flexure as $\mathrm{k_\theta = EI_f/L_f}$, where $E$ is the material's modulus of elasticity, $I_f$ is the area moment of inertia of the flexure, and $L_f$ is the length of the flexure. Similarly, combining Hooke's law ($\sigma = \mathrm{E}\epsilon$, where σ is stress and ε is strain), the axial stress equation ($\sigma = \mathrm{F/A}$, where $F$ is the axial force and $A$ is the cross-sectional area), and the spring stiffness equation ($\mathrm{F = k_A x}$, where $k_A$ is the linear spring stiffness) gives the axial stiffness of the small-length flexure as $\mathrm{k_A = EA_f/L_f}$, where $A_f$ is the cross-sectional area of the flexure. These relationships show that force scales with size by the power of 2 for both the torsional spring approximation ($F = \frac{\tau}{x} = \frac{k_\theta \Delta\theta}{x} = \frac{EI_f \Delta\theta}{xL}$) and the axial spring approximation ($F = k_A x = \frac{EA_f x}{L}$), as $I_f$ scales with size by the power of 4, $x$ and $L$ scale with size by the power of 1, and $A_f$ scales with size by the power of 2. The energy scales with size by the power of 3 for both the torsional and axial spring approximations, as energy is the integral of the force-displacement curve and displacement scales with size by the power of 1. These relations also show that the spring stiffness scales linearly with the elastic modulus, suggesting that the force and energy barriers will also scale linearly with the elastic modulus.

Transformation in multiple directions

The bistable unit cell is designed to enable transformation in the direction perpendicular to the top and bottom faces (fig. S7A). For example, in Figure 1E, Figure 2E-F, and Figure 3B, we demonstrate axial expansion and contraction of a cylindrical structure, where the extension/compression motion is enabled by arranging the unit cells with their transformation directions parallel to the cylinder axis. Then, in Figure 3C, we demonstrate a tilt stage, where the bending motion is enabled by arranging two metamaterials with their transformation directions parallel to each other and tangential to the desired bending motion. Because the two metamaterials in the tilt stage are not connected, transformation of one metamaterial enables bending motion while transformation of both metamaterials enables extension/compression motion.

Transformation in other directions can be similarly achieved by arranging the transformation direction of the metamaterial's unit cells to be parallel to the desired motion. In fig. S7, we show a sphere-like structure capable of three-dimensional transformation. The three-dimensional transformation is achieved by arranging metamaterials along the edges of a regular octahedron and orienting the transformation directions parallel to the x, y, or z-direction. For example, as shown in fig. S7D, transformation in the x-direction is achieved using four metamaterials in the y-z plane that have their transformation direction parallel to the x-axis. This concept can be expanded to create structures with higher complexity or improved shape fidelity using different polyhedra (e.g., a truncated icosahedron would form a shape that is even more spherical).

The three-dimensional shape in fig. S7D is created with metamaterials that have an expansion ratio of 1.93 (corresponding to a unit cell with $\theta = 60°$). The structure shown in the illustrations has a volumetric expansion ratio $\lambda_v$ of 1.76, where $\lambda_v$ is calculated as the volume of the structure in state [000] divided by the volume in state [111]. The $\lambda_v$ can be increased in at least two ways: first, by increasing $\lambda$ for the metamaterial unit cells (e.g., by using a larger $\theta$), and second, by increasing the ratio of the structure's surface area that is made from metamaterials (e.g., by using a polyhedron with more edges). Indeed, a sphere entirely made from metamaterials would have its circumference increase by $\lambda$, its surface area increase by $\lambda^2$, and its volume increase by $\lambda^3$ due to the geometric relationships between the circumference, surface area, and volume of a sphere. Using these relations, we can see that the $\lambda_v$ for the structure in fig. S7 is lower than the theoretical maximum for a sphere ($\lambda_{v,max} = \lambda^3 = 7.2$) with the same $\lambda$. Additionally, as discussed in the manuscript, the stability of the structure in state [000] can increase by increasing $\theta$, *R*, and *t/L*.

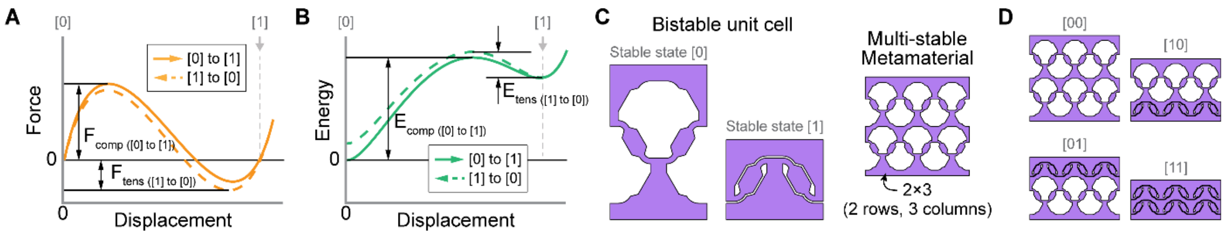


**Fig. S1.**

Multi-stability and metamaterial terminology. (A-B) Force-displacement and energy-displacement curves for a generic bistable metamaterial. If a geometry is bistable, the force curve will cross below zero and the energy curve will have a local minimum at [1]. As shown in A, the force barrier in compression ($F_{comp}$) is the maximum of the force curve from [0] to [1], and the force barrier in tension ($F_{tens}$) is the minimum of the force curve from [1] to [0]. As shown in B, the energy barrier in compression ($E_{comp}$) is the maximum of the energy curve from [0] to [1], and the energy barrier in tension ($E_{tens}$) is the difference between the maximum of the energy curve from [1] to [0] and the local minimum at [1]. (C) A multi-stable metamaterial is composed of bistable unit cells designed to be stable in state [0] and state [1]. (D) The number of distinct stable states for a metamaterial is $2^N$, where $N$ is the number of independent parallel arrangements of unit cells (e.g., number of rows). For example, we show a metamaterial with two rows of three unit cells each (denoted 2×3) which has four distinct stable states.

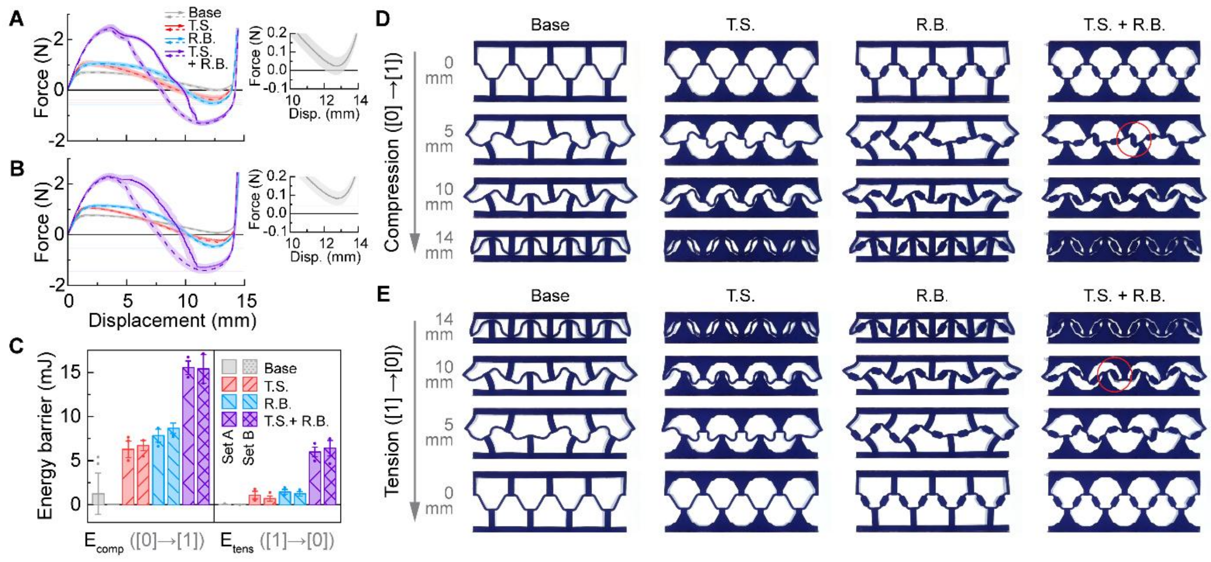


**Fig. S2.**

**Geometry modifications to enhance multi-stability.** (**A-B**) Force-displacement curves of two sets of 1×4 metamaterials, where the shaded regions around data show one standard deviation from eight experiments. Both sets have the same geometry (one model of each unit cell type) and have elastic moduli of 830 kPa (A) and 813 kPa (B), respectively. The insets show that the mean force of the Base geometry does not cross zero during compression. (**C**) Energy barriers in compression ($E_{comp}$) and tension ($E_{tens}$), corresponding to the data in (A) and (B), where the error bars show one standard deviation from eight experiments, and outliers are indicated by data points. The results show the synergistic increase in $E_{tens}$ for the geometry with both trapezoidal supporting segments ("T.S.") and reinforced beams ("R.B."). The high error in $E_{comp}$ for set A is due to only two of the eight experiments that crossed zero force. (**D-E**) Images showing the 1×4 metamaterials of each geometry in (A) at four displacements in compression (D) and tension (E) during tensile testing. A comparison between images shows that the T.S. geometry decreases deflection in the supporting segments, and the R.B. geometry constrains the beam's bending behaviors. By designing the metamaterial with both T.S. and R.B., the highly constrained unit cell motion causes beams to become temporarily locked in orientations that cause compression of the reinforced sections of the beams (e.g., see red circles in D and E), which causes hysteresis in the force curves and increases the energy required to transition between states in both loading directions.

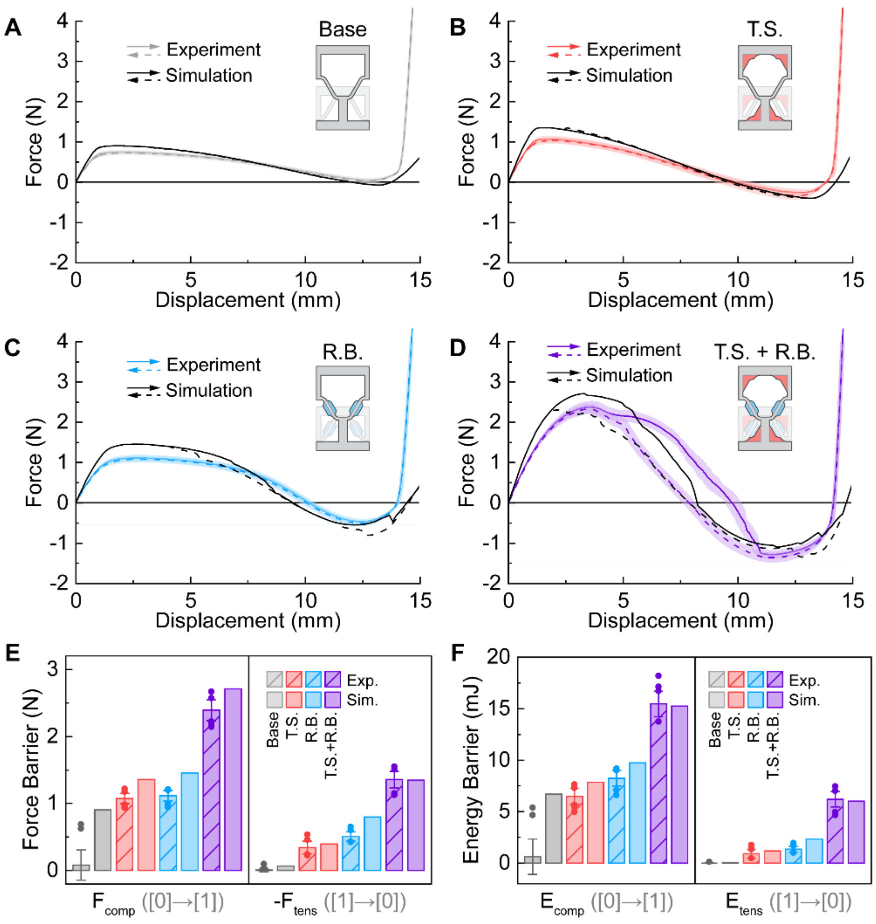


**Fig. S3.**

**Comparison between experiment and FE simulation results.** (**A-D**) Force-displacement curves in compression (solid) and tension (dashed) for 1×4 metamaterials with unit cells having the Base geometry (A), T.S. geometry (B), R.B. geometry (C), and T.S. + R.B. geometry (D). The experimental data are the same as shown in fig. S2, where the lines indicate the mean and the shaded regions show one standard deviation for 16 experiments. The simulation data are in black. (**E-F**) Force barriers (E) and energy barriers (F) for each geometry, where the experimental data have crosshatched bars. The error bars represent one standard deviation across 16 experiments, and outliers are indicated by data points. To simplify the representation and make all data positive in the plot, the $F_{tens}$ is multiplied by -1. A comparison between the experiment and FE simulation results shows that the simulations tend to slightly overpredict the force and energy barriers, which may be attributed to the assumptions in the FE simulations. Moreover, the results show the FE simulations accurately capture the dominant behaviors and the differences in the stability between the metamaterial designs. The large difference between the experimental and simulation $F_{comp}$ and $E_{comp}$ for the Base geometry can be attributed to 14 of the 16 experiments that did not cross zero force and thus have $F_{comp}$ = 0 and $E_{comp}$ = 0.

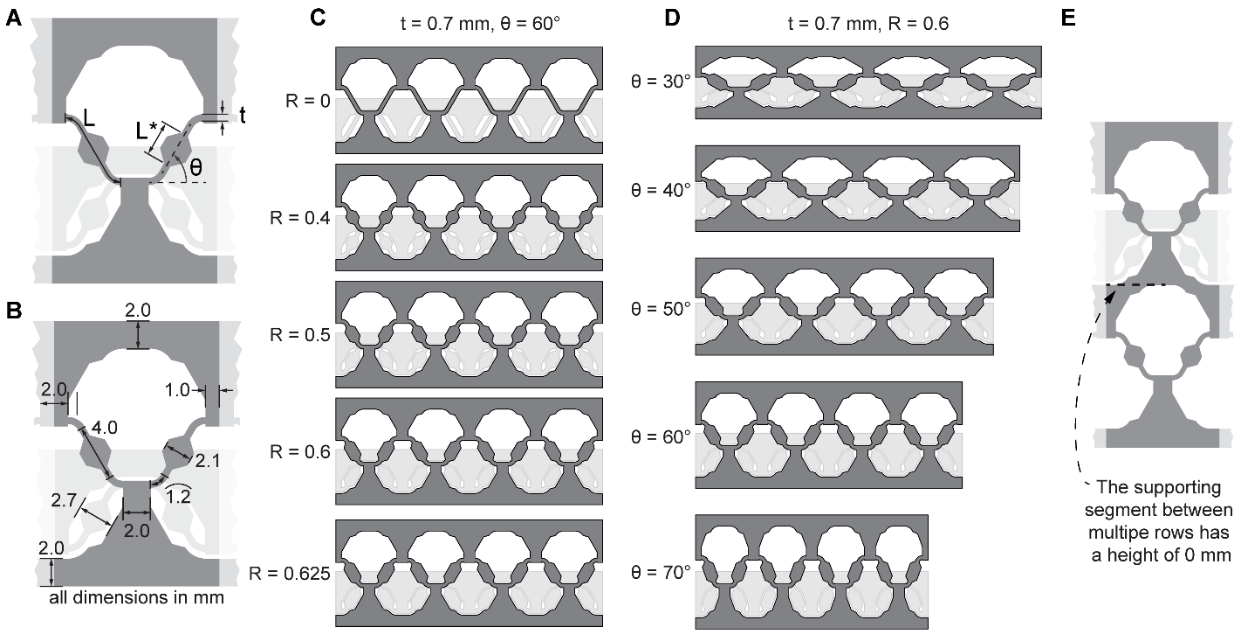


**Fig. S4.**

**Dimensions and profiles of 1×4 metamaterials used in the parameter studies**. **A-B**) Schematics show the unit cells in state [0] of the two-dimensional 1×4 models used in the FE simulations, where (A) shows the design variables used in the parameter studies, and (B) shows the other dimensions. **C**) Profiles show the effect of changing *R* on the geometry. The effect of changing *t* is not shown. **D**) Profiles show the effect of changing θ on the geometry, where θ also affects the supporting segment geometry to maintain the length *L* and the compactness in the closed state. Changing θ also affects the expansion ratio (λ), where λ = 1.66, 1.78, 1.87, 1.93, and 1.98 for designs with θ = 30°, 40°, 50°, 60°, and 70°, respectively. The effect of changing *t* is not shown. (**E**) To increase the expansion ratio for the designs with multiple rows, we chose to reduce the height of the horizontal portion of the supporting segment to 0 mm.

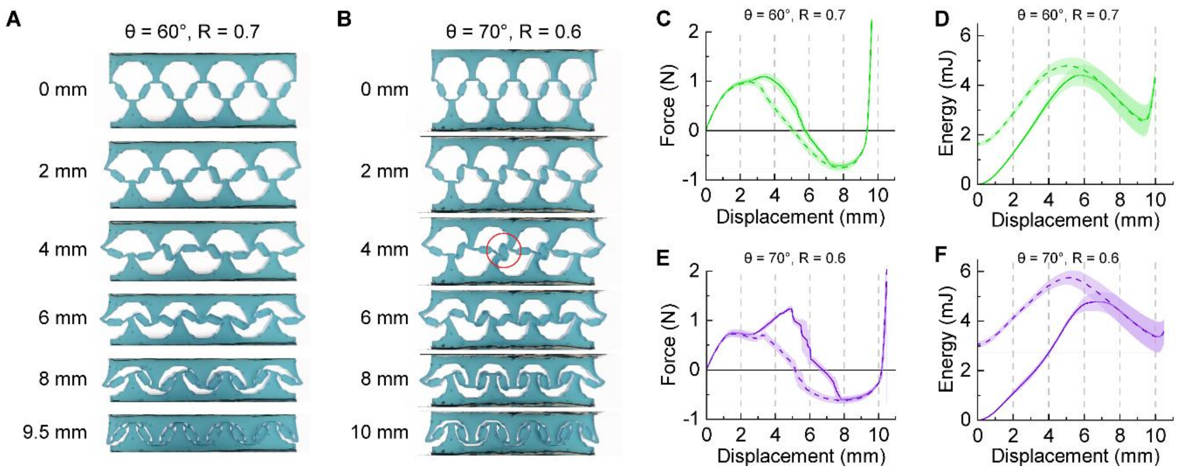


**Fig. S5.**

**Soft, multi-stable metamaterials that exceed the kinematic feasibility limit.** (**A-B**) Images showing the relative deformations of 1×4 metamaterials at six displacements during loading. Both metamaterials have $t$ = 0.7 mm. In (A), the value of $R$ = 0.7 is greater than the theoretical limit of 0.65 given by $R < \cos(\theta_{eff})$, where $\theta_{eff}$ = 57.6° includes the curved ends of the beam. For the design in (A), the higher value of $R$ is achieved by extending the reinforced section into the curved portions of the beam. In (B), the value of $R$ = 0.6 is greater than the limit of 0.54, where $\theta_{eff}$ = 49.2°. (**C** to **F**) Data from tensile testing showing the force curves (C, E) and energy curves (D, F) of the metamaterials in A (C-D) and B (E-F). The shaded regions around data show one standard deviation across five experiments. The hysteresis in the force-displacement curves (C and E), and the non-zero energy at the end of the loading cycle (D and F) can be attributed to energy dissipation within the metamaterial due to the highly constrained geometry. For the metamaterial in (B), the higher values of $R$ and θ cause geometric contact to occur during loading (e.g., indicated by the red circle in B), which increases the force required to transition to state [1] (see the increase in force near displacement = 4 mm in E).

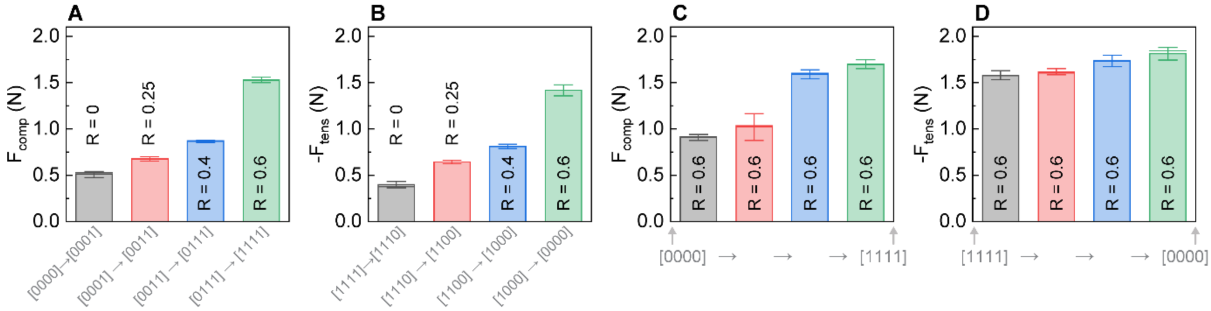


**Fig. S6.**

**Force barriers for 4×8 cylindrical metamaterials.** Plots show the force barriers in the order of transformation (black bar: first row to transform, green bar: last row to transform). The error bars represent one standard deviation across five experiments. To simplify the representation and make all data positive in the plots, the $F_{tens}$ is multiplied by -1. (**A-B**) Force barriers in compression (A) and tension (B) for the 4×8 cylindrical metamaterial with a different $R$ in each row. Due to the different $R$ in each row, the rows transform in a predictable order, where the top row ($R$ = 0) transforms first in both directions and the bottom row ($R$ = 0.6) transforms last in both directions. (**C-D**) Force barriers in compression (C) and tension (D) for the 4×8 cylindrical metamaterial with $R$ = 0.6 in each row. Due to the similarity between rows, the order of transformation is not predictable and the x-axis labels only indicate the initial and final states of the metamaterial. The relatively lower values of $F_{comp}$ for the first two transformations in (C) can be attributed to differences in the boundary conditions as the deformation occurs across multiple rows. In some cases for the metamaterial with $R$ = 0.6 in each row, the closeness in the stability between the rows and the flexibility of the supporting segments between rows caused the transformation in compression to occur across rows.

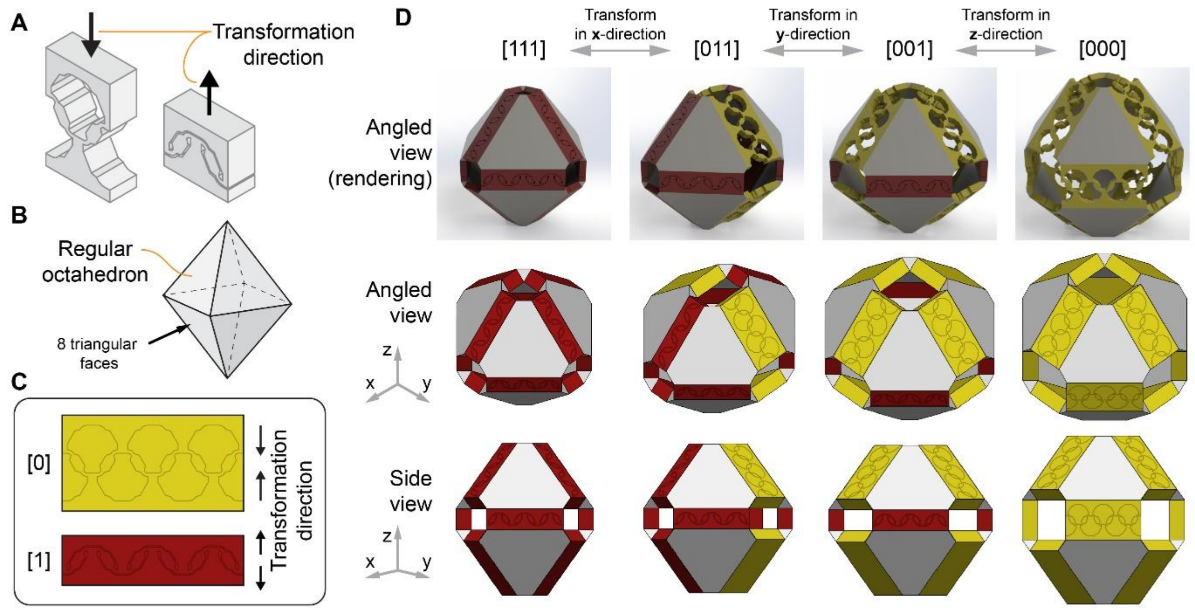


**Fig. S7.**

**Assembling metamaterials for three-dimensional transformation.** (**A**) The bistable unit cell is designed to enable transformation in the direction perpendicular to the top and bottom faces. (**B**) A regular octahedron has eight edges and 12 triangular faces. (**C-D**) Renderings and illustrations of a structure made from 12 metamaterials. In the renderings, the metamaterials are colored red in state [1] and yellow in state [0] to more easily distinguish between the configurations. Similarly, in the illustrations, the metamaterials are represented by yellow rectangular prisms in state [0], and red rectangular prisms in state [1]. The metamaterials are connected with gray segments that represent the polyhedron faces. The metamaterials are arranged along the edges of a regular octahedron and oriented with their transformation directions parallel to the x, y, or z-direction to enable expansion in all three orthogonal directions, where four metamaterials are in each plane (e.g., transformation in the x-direction is enabled by the four metamaterials arranged in the y-z plane).

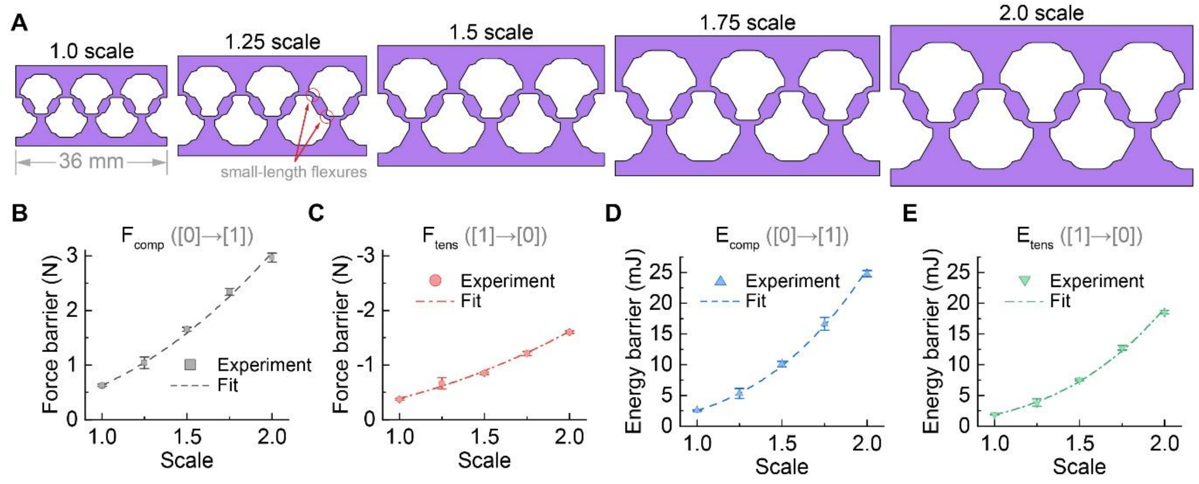


**Fig. S8.**

Effect of scale on the force and energy barriers of 1×3 metamaterials. (A) The metamaterial is scaled uniformly in all directions at 1, 1.25, 1.5, 1.75, and 2× scale. The 1× scale metamaterial has $t$ = 0.7 mm, θ = 60°, $R$ = 0.6, a total width of 36 mm, and an out-of-plane thickness of 5 mm. (B-E) Tensile testing was used to measure the force barriers (B-C) and energy barriers (D-E) of the experimental specimens. Error bars show the standard deviation across five experiments. An exponential fit (dashed lines) was calculated for each data set as follows, with the correlation coefficient (R) reported for each fit. $F_{comp}$: $y = 0.63x^{2.28}$, R = 0.99; $F_{tens}$: $y = -0.39x^{2.05}$, R = 0.98; $E_{comp}$: $y = 2.57x^{3.31}$, R = 0.99; $E_{tens}$: $y = 1.88x^{3.35}$, R = 0.99.

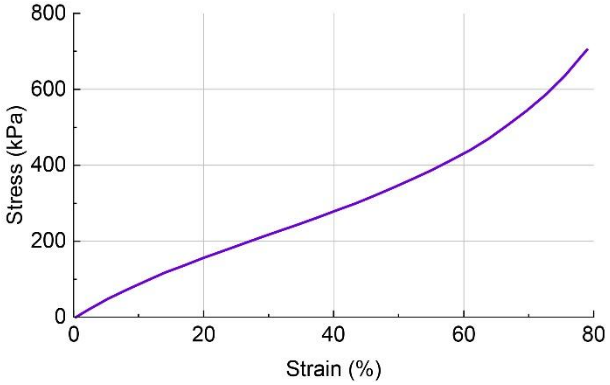


**Fig. S9.**

**Experimental stress-strain curve in uniaxial tension for the cured silicone used in the experiments.** The stress-strain curve was used to create the Marlow material model used in the FE simulations. A linear fit to the data is used to estimate the Young's Modulus at 809 kPa.

| | Geometry: | $E_{comp}$ (mJ) Base | T.S. | R.B. | T.S.+R.B | $E_{tens}$ (mJ) Base | T.S. | R.B. | T.S.+R.B |
|---|---|---|---|---|---|---|---|---|---|
| Set A | Experiment 1 | 0 | 5.880 | 8.458 | 16.238 | 0 | 1.437 | 1.277 | 5.637 |
| | Experiment 2 | 0 | 6.803 | 8.506 | 16.699 | 0 | 0.821 | 1.216 | 4.950 |
| | Experiment 3 | 0 | 4.977 | 6.611 | 15.094 | 0.013 | 1.761 | 1.954 | 6.295 |
| | Experiment 4 | 0 | 7.248 | 7.613 | 15.491 | 0 | 0.619 | 1.543 | 5.924 |
| | Experiment 5 | 5.376 | 5.479 | 7.673 | 15.319 | 0.030 | 1.412 | 1.485 | 6.097 |
| | Experiment 6 | 4.667 | 7.641 | 7.065 | 14.414 | 0.135 | 0.472 | 1.741 | 6.929 |
| | Experiment 7 | 0 | 5.631 | 8.654 | 14.890 | 0 | 1.335 | 1.103 | 6.175 |
| | Experiment 8 | 0 | 6.534 | 8.107 | 16.021 | 0 | 0.867 | 1.298 | 5.828 |
| Set B | Experiment 1 | 0 | 7.093 | 7.697 | 16.448 | 0 | 0.677 | 1.703 | 6.003 |
| | Experiment 2 | 0 | 5.467 | 8.451 | 14.310 | 0 | 1.371 | 1.357 | 6.707 |
| | Experiment 3 | 0 | 6.357 | 7.841 | 17.114 | 0 | 0.865 | 1.567 | 5.785 |
| | Experiment 4 | 0 | 7.286 | 9.030 | 18.183 | 0 | 0.544 | 1.078 | 4.670 |
| | Experiment 5 | 0 | 6.786 | 9.020 | 14.461 | 1.09E-05 | 0.585 | 1.098 | 7.464 |
| | Experiment 6 | 0 | 6.853 | 9.152 | 13.730 | 0 | 0.553 | 1.036 | 7.020 |
| | Experiment 7 | 0 | 6.719 | 9.171 | 14.423 | 0 | 0.586 | 1.012 | 6.765 |
| | Experiment 8 | 0 | 7.011 | 8.882 | 14.415 | 0 | 0.475 | 1.121 | 6.809 |

**Table S1.**

**Experimental energy barriers for the 1×4 metamaterials with Base, T.S., R.B., and T.S. + R.B. geometries.** The values of $E_{comp}$ = 0 and $E_{tens}$ = 0 in the Base geometry are due to the force-displacement curve not crossing zero in the given direction. The data in this table are used in the plots in fig. S2C and fig. S3F. In fig. S3F, data from set A and set B are combined.

| | | $F_{comp}$ (N) | | | | $F_{tens}$ (N) | | | |
|---|---|---|---|---|---|---|---|---|---|
| | **Geometry:** | **Base** | **T.S.** | **R.B.** | **T.S.+R.B** | **Base** | **T.S.** | **R.B.** | **T.S.+R.B** |
| Set A | Experiment 1 | 0 | 1.051 | 1.134 | 2.572 | 0 | 0.459 | 0.496 | 1.267 |
| | Experiment 2 | 0 | 1.140 | 1.143 | 2.668 | 0 | 0.343 | 0.476 | 1.151 |
| | Experiment 3 | 0 | 0.934 | 0.944 | 2.425 | 0.026 | 0.538 | 0.651 | 1.367 |
| | Experiment 4 | 0 | 1.176 | 1.050 | 2.517 | 0 | 0.268 | 0.559 | 1.300 |
| | Experiment 5 | 0.692 | 0.985 | 1.059 | 2.492 | 0.038 | 0.459 | 0.542 | 1.334 |
| | Experiment 6 | 0.625 | 1.217 | 0.992 | 2.226 | 0.100 | 0.226 | 0.602 | 1.451 |
| | Experiment 7 | 0 | 1.001 | 1.154 | 2.375 | 0 | 0.434 | 0.442 | 1.342 |
| | Experiment 8 | 0 | 1.096 | 1.098 | 2.470 | 0 | 0.326 | 0.496 | 1.293 |
| Set B | Experiment 1 | 0 | 1.126 | 1.067 | 2.476 | 0 | 0.292 | 0.604 | 1.354 |
| | Experiment 2 | 0 | 0.951 | 1.142 | 2.359 | 0 | 0.457 | 0.517 | 1.460 |
| | Experiment 3 | 0 | 1.050 | 1.076 | 2.436 | 0 | 0.335 | 0.570 | 1.268 |
| | Experiment 4 | 0 | 1.134 | 1.201 | 2.509 | 0 | 0.242 | 0.442 | 1.126 |
| | Experiment 5 | 0 | 1.076 | 1.201 | 2.176 | 8.30E-04 | 0.279 | 0.449 | 1.551 |
| | Experiment 6 | 0 | 1.084 | 1.209 | 2.142 | 0 | 0.275 | 0.432 | 1.509 |
| | Experiment 7 | 0 | 1.067 | 1.209 | 2.216 | 0 | 0.292 | 0.426 | 1.471 |
| | Experiment 8 | 0 | 1.092 | 1.184 | 2.208 | 0 | 0.251 | 0.451 | 1.476 |

**Table S2.**

**Experimental force barriers for the 1×4 metamaterials with Base, T.S., R.B., and T.S. + R.B. geometries.** The values of $F_{comp}$ = 0 and $F_{tens}$ = 0 in the "Base" geometry are due to the force-displacement curve not crossing zero in the given direction. The data in this table are used in the plot in fig. S3F. In the plot, the data from set A and set B are combined.

| | $E_{tens}$ (mJ) ([11]→[10]) | $E_{tens}$ (mJ) ([10]→[00]) |
|---|---|---|
| Experiment 1 | 0.630 | 3.587 |
| Experiment 2 | 0.604 | 3.407 |
| Experiment 3 | 0.531 | 3.401 |
| Experiment 4 | 0.520 | 3.393 |
| Experiment 5 | 0.620 | 3.598 |

**Table S3.**

**Experimental energy barriers in tension for the 2×8 cylindrical metamaterial with *R* = 0.6 in the bottom row and *R* = 0 in the top row.** The data in this table are used to create the plot in Fig. 2D.

| | | $F_{comp}$ (N) | | | | $F_{tens}$ (N) | | | |
|---|---|---|---|---|---|---|---|---|---|
| | | **first row** | **second row** | **third row** | **fourth row** | **first row** | **second row** | **third row** | **fourth row** |
| Different R in each row | Experiment 1 | 0.534 | 0.710 | 0.875 | 1.525 | -0.367 | -0.642 | -0.829 | -1.466 |
| | Experiment 2 | 0.525 | 0.671 | 0.851 | 1.484 | -0.376 | -0.642 | -0.843 | -1.484 |
| | Experiment 3 | 0.534 | 0.679 | 0.859 | 1.518 | -0.375 | -0.612 | -0.789 | -1.417 |
| | Experiment 4 | 0.475 | 0.676 | 0.882 | 1.559 | -0.444 | -0.664 | -0.811 | -1.384 |
| | Experiment 5 | 0.470 | 0.651 | 0.872 | 1.553 | -0.426 | -0.650 | -0.786 | -1.334 |
| $R$ = 0.6 in each row | Experiment 1 | 0.876 | 0.839 | 1.534 | 1.694 | -1.618 | -1.650 | -1.822 | -1.818 |
| | Experiment 2 | 0.876 | 0.917 | 1.540 | 1.697 | -1.636 | -1.655 | -1.734 | -1.842 |
| | Experiment 3 | 0.948 | 1.034 | 1.642 | 1.774 | -1.575 | -1.592 | -1.651 | -1.693 |
| | Experiment 4 | 0.917 | 1.129 | 1.600 | 1.650 | -1.517 | -1.603 | -1.716 | -1.851 |
| | Experiment 5 | 0.918 | 1.182 | 1.626 | 1.676 | -1.551 | -1.588 | -1.754 | -1.854 |

**Table S4.**

**Experimental energy barriers in tension for the 4×8 cylindrical metamaterials.** The data in this table are used in the plots in fig. S6.

| | | $F_{comp}$ (N) | $F_{tens}$ (N) | $E_{comp}$ (mJ) | $E_{tens}$ (mJ) |
|---|---|---|---|---|---|
| 1× scale | Experiment 1 | 0.656 | -0.348 | 2.724 | 1.982 |
| | Experiment 2 | 0.634 | -0.369 | 2.614 | 1.940 |
| | Experiment 3 | 0.592 | -0.403 | 2.427 | 1.769 |
| | Experiment 4 | 0.626 | -0.370 | 2.597 | 1.928 |
| | Experiment 5 | 0.642 | -0.378 | 2.463 | 1.891 |
| 1.25× scale | Experiment 1 | 1.093 | -0.621 | 5.907 | 4.128 |
| | Experiment 2 | 0.826 | -0.864 | 3.817 | 2.657 |
| | Experiment 3 | 1.100 | -0.646 | 5.113 | 3.937 |
| | Experiment 4 | 1.067 | -0.621 | 5.765 | 4.140 |
| | Experiment 5 | 1.121 | -0.570 | 6.028 | 4.397 |
| 1.5× scale | Experiment 1 | 1.700 | -0.847 | 9.740 | 7.569 |
| | Experiment 2 | 1.692 | -0.859 | 10.568 | 7.421 |
| | Experiment 3 | 1.647 | -0.872 | 9.508 | 7.450 |
| | Experiment 4 | 1.642 | -0.813 | 10.715 | 7.844 |
| | Experiment 5 | 1.601 | -0.855 | 9.986 | 7.528 |
| 1.75× scale | Experiment 1 | 2.238 | -1.283 | 15.666 | 12.074 |
| | Experiment 2 | 2.392 | -1.191 | 16.030 | 13.051 |
| | Experiment 3 | 2.408 | -1.183 | 18.685 | 13.222 |
| | Experiment 4 | 2.342 | -1.204 | 16.262 | 12.812 |
| | Experiment 5 | 2.317 | -1.191 | 16.582 | 12.710 |
| 2.0× scale | Experiment 1 | 3.018 | -1.576 | 25.316 | 18.973 |
| | Experiment 2 | 2.858 | -1.643 | 24.724 | 18.074 |
| | Experiment 3 | 2.892 | -1.600 | 25.421 | 18.492 |
| | Experiment 4 | 3.076 | -1.584 | 24.308 | 18.780 |
| | Experiment 5 | 3.017 | -1.618 | 24.199 | 18.418 |

**Table S5.**

**Experimental force and energy barriers for the 1×3 metamaterials across scales.** The data in this table are used in the plots in Fig S7.

| | | Pre-stressor | | | | Post-stressor | | | |
|---|---|---|---|---|---|---|---|---|---|
| | | $E_{comp}$ (mJ) | | $E_{tens}$ (mJ) | | $E_{comp}$ (mJ) | | $E_{tens}$ (mJ) | |
| | | **first row** | **second row** | **first row** | **second row** | **first row** | **second row** | **first row** | **second row** |
| Fig. 5C | Experiment 1 | 6.595 | 6.978 | 4.496 | 3.793 | 4.930 | 7.297 | 5.724 | 3.738 |
| | Experiment 2 | 6.471 | 6.963 | 4.460 | 3.627 | 6.299 | 7.561 | 5.039 | 3.061 |
| | Experiment 3 | 6.469 | 7.374 | 4.064 | 3.572 | 6.241 | 7.532 | 4.944 | 2.362 |
| | Experiment 4 | 6.669 | 7.051 | 3.934 | 3.878 | 5.753 | 7.737 | 4.781 | 4.185 |
| | Experiment 5 | 6.543 | 6.460 | 4.088 | 3.897 | 6.199 | 7.486 | 4.566 | 3.983 |
| Fig. 5F | Experiment 1 | 6.723 | 7.158 | 5.485 | 4.191 | 3.962 | 4.405 | 2.278 | 3.069 |
| | Experiment 2 | 6.730 | 7.237 | 5.620 | 2.355 | 4.079 | 4.082 | 2.321 | 3.055 |
| | Experiment 3 | 6.264 | 6.990 | 5.312 | 3.927 | 4.093 | 4.250 | 2.842 | 1.637 |
| | Experiment 4 | 5.991 | 6.868 | 5.028 | 4.266 | 3.613 | 4.932 | 2.714 | 1.658 |
| | Experiment 5 | 6.582 | 6.782 | 4.580 | 3.808 | 3.877 | 4.789 | 2.662 | 1.525 |
| Fig. 5I | Experiment 1 | 7.097 | 6.844 | 4.734 | 4.227 | 7.964 | 8.915 | 4.829 | 2.281 |
| | Experiment 2 | 6.966 | 6.730 | 4.671 | 4.340 | 7.804 | 8.097 | 4.855 | 4.099 |
| | Experiment 3 | 6.656 | 7.163 | 4.610 | 4.401 | 6.605 | 7.273 | 6.067 | 3.112 |
| | Experiment 4 | 6.677 | 7.235 | 4.680 | 4.301 | 6.672 | 6.937 | 5.096 | 4.455 |
| | Experiment 5 | 6.765 | 6.857 | 4.643 | 4.330 | 6.365 | 7.081 | 5.805 | 4.643 |
| Fig. 5L | Experiment 1 | 4.441 | 5.291 | 3.965 | 3.595 | 4.749 | 5.229 | 4.928 | 3.191 |
| | Experiment 2 | 4.474 | 5.316 | 3.923 | 3.552 | 5.191 | 5.381 | 4.620 | 2.631 |
| | Experiment 3 | 4.488 | 5.387 | 3.785 | 3.388 | 4.365 | 5.314 | 4.687 | 3.059 |
| | Experiment 4 | 4.619 | 5.709 | 3.867 | 3.818 | 4.452 | 5.253 | 4.717 | 2.972 |
| | Experiment 5 | 4.721 | 5.200 | 3.903 | 3.604 | 4.241 | 5.313 | 4.670 | 2.881 |

**Table S6.**

**Experimental energy barriers for the 2×8 cylindrical metamaterials before and after the adverse stressors.** The data in this table are used in the plots in Fig 5.

| Model location | Geometry | *t* (mm) | *R* | θ (deg.) | Out-of-plane thickness (mm) | Other dimensions (scale of Fig S4) | Note |
|---|---|---|---|---|---|---|---|
| Fig. 1 C-D, Fig S2, S3 | Planar, 1×4 | 1.05 | 0 and 0.6 | 60 | 7.5 | 1.5× | * |
| Fig. 1 E | Cylinder, 4×8 | 0.595 | 0.6, 0.4, 0.25, 0 | 60 | 4.25 | 0.85× | |
| Fig. 2 B-C | Planar, 1×4 | 0.5, 0.6, 0.7, 0.8 | 0, 0.4, 0.5, 0.6, 0.625 | 30, 40, 50, 60, 70 | 5 | 1× | |
| Fig. 2 D-E | Cylinder, 2×8 | 0.595 | 0.6, 0 | 60 | 4.25 | 0.85× | |
| Fig 2 F-G | Cylinder, 4×8 | 0.595 | 0.6, 0.4, 0.25, 0 | 60 | 4.25 | 0.85× | |
| Fig 3 A | Cylinder, 2×8 | 0.595 | 0.6 | 60 | 4.25 | 0.85× | |
| Fig 3 B | Cylinder, 2×8 | 0.595 | 0.6, 0 | 60 | 4.25 | 0.85× | |
| Fig 3 C | Planar, 1×3 | 0.595 | 0.6 | 60 | 4.25 | 0.85× | |
| Fig 3 D | Planar, 1×3 | 0.8 | 0.625 | 60 | 6.38 | 0.85× | |
| Fig 4 A-B | Cylinder, 2×8 | 0.595 | 0.6 | 60 | 4.25 | 0.85× | |
| Fig 4 C-F | Cylinder, 4×8 | 0.595 | 0.6, 0.4, 0.25, 0 | 60 | 4.25 | 0.85× | |
| Fig 5 | Cylinder, 2×8 | 0.595 | 0.6 | 60 | 4.25 | 0.85× | |
| Fig S5 A | Planar, 1×4 | 0.7 | 0.7 | 60 | 5 | 1× | |
| Fig S5 B | Planar, 1×4 | 0.7 | 0.6 | 70 | 5 | 1× | |
| Fig S6 A-B | Cylinder, 4×8 | 0.595 | 0.6, 0.4, 0.25, 0 | 60 | 4.25 | 0.85× | |
| Fig S6 C-D | Cylinder, 4×8 | 0.595 | 0.6 | 60 | 4.25 | 0.85× | |
| Fig S7 (1× scale) | Planar, 1×3 | 0.7 | 0.6 | 60 | 5 | 1× | † |

**Table S7.**

**The geometry and dimensions of the metamaterials used in this study.** (*) The Base and T.S. designs have $R = 0$, the R.B. and T.S. + R.B. designs have $R = 0.6$, and the Base and R.B. designs do not have the "T.S." geometry in the supporting segments. (†) The 1.25×, 1.5×, 1.75×, and 2.0× models are scaled uniformly in all directions.

**Movie S1.**

Magnetic field-induced transformation of two 4×8 cylindrical metamaterials.

**Movie S2.**

High-speed video of the 4×8 cylindrical metamaterials transformation with external magnetic fields.

**Movie S3.**

Magnetic field-induced transformation to deploy electronics from a surface.

**Movie S4.**
Magnetic field-induced transformation of a tilt stage between four stable configurations.

**Movie S5.**
Flow control using 1×3 metamaterials.

**Movie S6.**
Magnetic field-induced transformation between approximately -20 and 100 °C.

**Movie S7.**
Stability and magnetic field-induced transformation in dynamic environments.

**Movie S8.**
Adverse stressor experiment: stretching.

**Movie S9.**
Adverse stressor experiment: blunt impact.

**Movie S10.**
Adverse stressor experiment: within simulated gastric fluid.

**Movie S11.**
Adverse stressor experiment: fire.
